# Mechanisms of Heme Utilization by *Francisella tularensis*

**DOI:** 10.1371/journal.pone.0119143

**Published:** 2015-03-10

**Authors:** Helena Lindgren, Lena Lindgren, Igor Golovliov, Anders Sjöstedt

**Affiliations:** Department of Clinical Microbiology, Clinical Bacteriology, and Laboratory for Molecular Infection Medicine Sweden (MIMS), Umeå University, Umeå, Sweden; Université Paris Descartes, FRANCE

## Abstract

*Francisella tularensis* is a highly virulent facultative intracellular pathogen causing the severe disease tularemia in mammals. As for other bacteria, iron is essential for its growth but very few mechanisms for iron acquisition have been identified. Here, we analyzed if and how *F*. *tularensis* can utilize heme, a major source of iron *in vivo*. This is by no means obvious since the bacterium lacks components of traditional heme-uptake systems. We show that SCHU S4, the prototypic strain of subspecies *tularensis*, grew in *vitro* with heme as the sole iron source. By screening a SCHU S4 transposon insertion library, 16 genes were identified as important to efficiently utilize heme, two of which were required to avoid heme toxicity. None of the identified genes appeared to encode components of a potential heme-uptake apparatus. Analysis of SCHU S4 deletion mutants revealed that each of the components FeoB, the siderophore system, and FupA, contributed to the heme-dependent growth. In the case of the former two systems, iron acquisition was impaired, whereas the absence of FupA did not affect iron uptake but led to abnormally high binding of iron to macromolecules. Overall, the present study demonstrates that heme supports growth of *F*. *tularensis* and that the requirements for the utilization are highly complex and to some extent novel.

## Introduction

*Francisella tularensis* is a highly virulent facultative intracellular pathogen causing the severe disease tularemia in mammals [1]. Four subspecies exist, two of which are of clinical importance; the highly virulent subspecies *tularensis* (type A), which causes disease with high mortality if untreated, and the less aggressive subspecies *holarctica* (type B), which despite its lower virulence, may cause serious illness in humans. Regardless of subspecies, it is highly contagious with an infectious dose of less than 10 bacteria and in the mouse model it reaches high bacterial numbers within a few days of infection [1]. In *vitro*, *F*. *tularensis* is a fastidious organism requiring rich media supplemented with iron and cysteine to grow. In view of its high pathogenicity and ability to rapidly cause lethal infection and the low free iron concentration in vivo (~ 10^-18^ M), *F*. *tularensis* must possess mechanisms that despite its inability of effective iron utilization in *vitro*, must allow effective iron utilization in *vivo*.

The aquatic habitat of *F*. *tularensis* plays an essential role for its life cycle, but it is normally spread via arthropods and causes disease in a vast number of mammals and proliferates in many different cell types. This implies that it must possess highly sophisticated means to acquire iron under the extremely variable conditions of its life cycle, but, surprisingly, the only mechanisms identified for iron acquisition are the siderophore system, *fsl*, and Ferrous iron transport system, *feo* and, in fact, the two systems appear to be the only ones mediating iron uptake in *F*. *tularensis* LVS [2,3,4,5]. Thus, some mechanisms behind its iron acquisition and regulation likely have not been identified.

The essential role of iron for almost all bacteria can be traced back all the way to the premicrobial world when iron and sulphur were abundant, leading to the use of iron-sulphur clusters for electron transfer. This is still evident today since iron has essential functions for many cellular functions such as electron transport, glycolysis, DNA synthesis, and defense against oxidative stress [6].

The maintenance of a low free iron concentration is an important innate immune mechanism to restrict the multiplication of an invading pathogen. This iron limitation has forced successful pathogens to evolve sophisticated systems that can exploit the available iron sources in the body. A common mechanism, present in many bacteria and in *F*. *tularensis*, is to secrete siderophores to chelate ferric iron from their surroundings [7]. The siderophore produced by *F*. *tularensis* is structurally very similar to rhizoferrin, a polycarboxylate siderophore produced by *Rhizopus* species [2]. A cluster of seven genes, denoted the *Francisella siderophore locus* (*fsl*), is required to produce the siderophore and to take up siderophore-bound iron. *F*. *tularensis* strains utilize FslA to synthesize the siderophore and virulent strains use FslE for uptake [8]. The siderophore enhances growth of *F*. *tularensis* on iron-limited media but an *fslA* deletion mutant in the type A strain SCHU S4 did not lead to attenuation in the mouse model [9]. Thus, type A strains have developed siderophore-independent mechanisms to effectively acquire iron during infection. A major virulence determinant of *F*. *tularensis* is encoded by *fupA* and the encoded protein appears to affect iron acquisition by both siderophore-dependent and -independent mechanisms [9,10]. FupA has no homologue in other bacteria but belongs to the same protein family as the siderophore receptor FslE. Its siderophore-independent mechanism is related to uptake of ferrous iron and iron homeostasis in the bacterial cell [9].

Siderophore-independent iron acquisition mechanisms, found mostly in pathogenic bacteria, serve to sequester iron from host proteins via specific high-affinity outer membrane receptors [11]. Host proteins utilized as a source of iron are for example transferrin, lactoferrin, ferritin, free heme, or heme-containing proteins, such as hemoglobin or hemopexin. Some bacteria also secrete hemophores, which can sequester free heme or heme from heme-containing proteins. *F*. *tularensis* does not, however, encode homologues of outer membrane receptors of other Gram-negative bacteria that recognize heme, hemoproteins or hemophores. Moreover, the bacterium lacks a homologue of TonB, a molecule necessary in most bacteria for the translocation of heme-bound molecules. Heme and hemoglobin are attractive sources of iron for any pathogen since they are present at both intracellular and extracellular sites during infection, *e*.*g*., free heme may be released from necrotic cells at the site of infection and thereby become available. In addition, liver and spleen, the primary sites of replication for *F*. *tularensis*, are important for the recycling of iron from heme of senescent red blood cells or by scavenging hemoglobin-haptoglobin complexes from the circulation [12]. After endocytosis and degradation of hemoglobin in the endocytic pathway, heme is transported into the cytosol and could thus be available to *F*. *tularensis*, which replicates in this compartment. Therefore, it would be logical if *F*. *tularensis* could utilize heme or hemoglobin since they are abundant sources of iron present at several locations that are part of the life cycle of the bacterium.

Heme may not only be a potential source of iron but may also pose a threat to the pathogen since it is a low-molecular, lipophilic molecule, which readily intercalates into membranes and, in addition, catalyzes the formation of reactive oxygen species and may therefore affect lipid bilayers and cause severe cell damage [13,14]. Therefore, expression of iron/heme uptake proteins during infection is tightly regulated in response to iron levels in the environment, often through the ferric uptake regulator (Fur). Although the role of Fur of *F*. *tularensis* has not been studied in detail, it is noteworthy that genes critical for iron homeostasis likely are controlled by Fur since the *fsl* operon and other operons involved in iron uptake have a Fur box in their promoter regions [2,3].

The aim of the present study was to explore if *F*. *tularensis* could utilize heme and tolerate its potential toxicity and also to identify the mechanism(s) behind the utilization and tolerance. We demonstrate that *F*. *tularensis* grew efficiently with heme as the sole iron source and identified novel factors important for the iron utilization and resistance to heme toxicity. In addition, we also show that the already described iron-acquisition systems of *F*. *tularensis* make important contributions to the heme-dependent iron utilization.

## Results

### Growth of *F*. *tularensis* and iron incorporation with heme as the iron source

**Fig 1:**
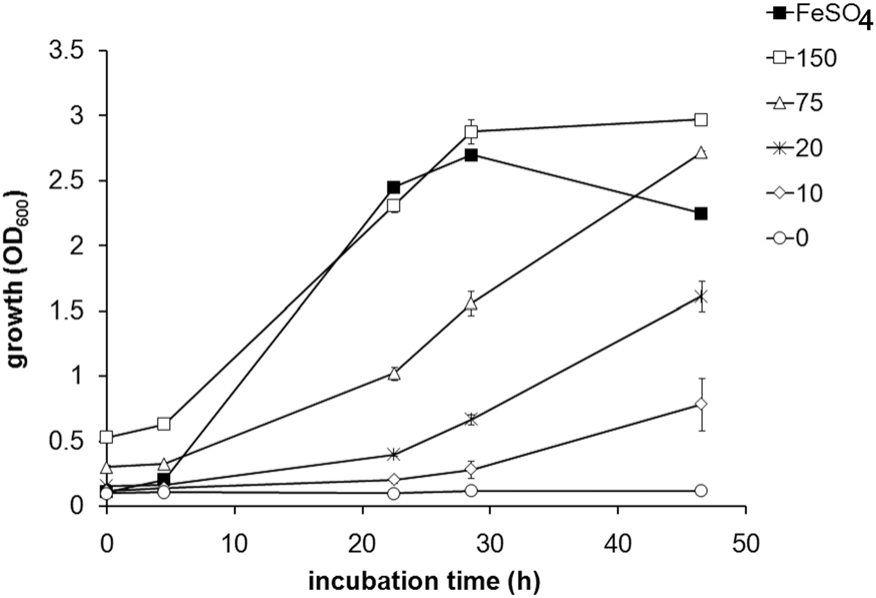
Growth of iron-depleted SCHU S4 in C-CDM supplemented with indicated concentrations of heme (open symbols) or 26 μM FeSO_4_ (closed symbol)

To characterize the requirements for the heme-utilization, the growth of SCHU S4 on various concentrations of heme was investigated. In the liquid medium C-CDM, the growth of SCHU S4 was strongly correlated to the concentration of heme (*P* < 0.01), Spearman’s rho 0.898 (Fig. 1). Notably, 150 μM of heme was required for SCHU S4 to grow as efficiently as in C-CDM supplemented with 26 μM FeSO_4_, containing equimolar amount of Fe as heme (Fig. 1). By use of the liquid heme-binding assay, it was found that iron-depleted SCHU S4 within 1 h had sequestered 21 ± 1.0, 34 ± 0.8 and 52 ± 3.0 nmol heme/OD from C-CDM supplemented with 75, 150, and 300 μM of heme, respectively.

**Fig 2:**
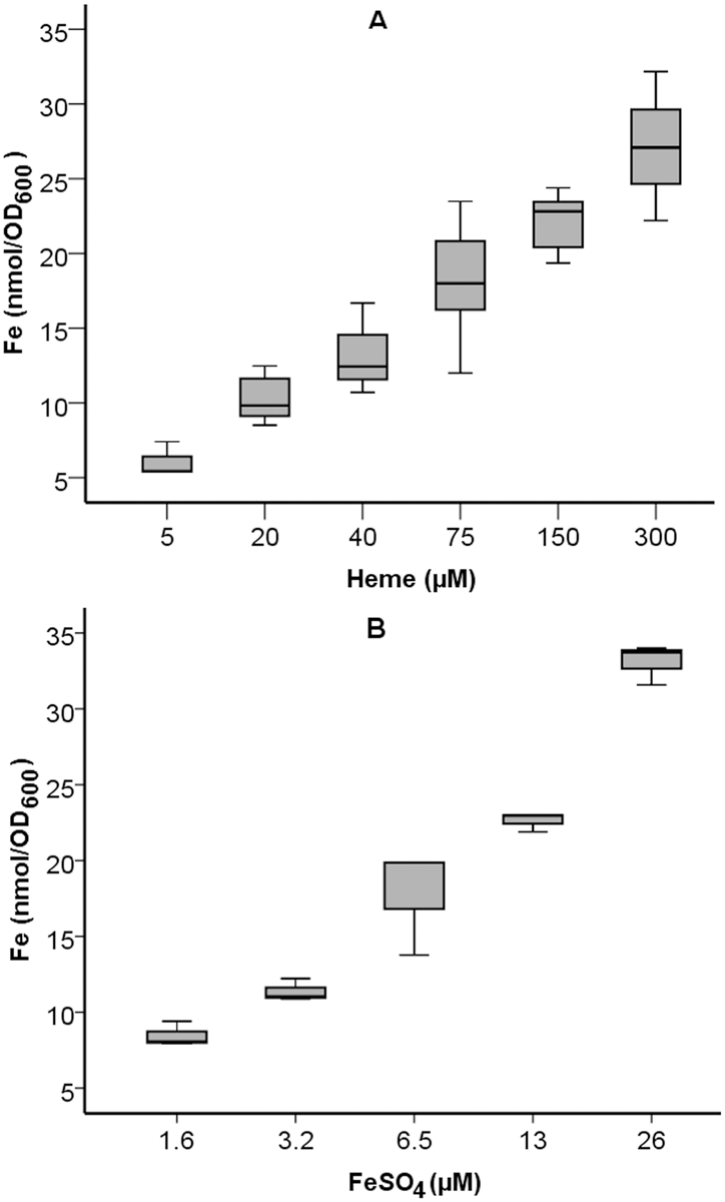
Iron incorporated by iron-depleted SCHU S4 during growth in C-CDM supplemented with increasing concentrations of A) heme or B) FeSO_4_. Samples for the ferrozine assay were withdrawn between 24 h and 48 h of incubation.

The amounts of iron incorporated by SCHU S4 growing at stationary phase in C-CDM supplemented with increasing concentrations of heme or FeSO_4_ was measured by the ferrozine assay. The incorporation was proportional to the heme concentration (*P* < 0.01; Spearman’s rho 0.834) (Fig. 2A) and the FeSO_4_ concentration (*P* < 0.01; Spearman’s rho 0.969) of the C-CDM (Fig. 2B). In view of the relatively poor growth of SCHU S4 with 20 μM heme, it was unexpected that SCHU S4 contained as much iron under these conditions (Fig. 2A) as after cultivation with 3.3 μM of FeSO_4_ (Fig. 2B), a concentration that supported growth of SCHU S4 to a similar extent as did 26 μM of FeSO_4_ (data not shown).

**Fig 3:**
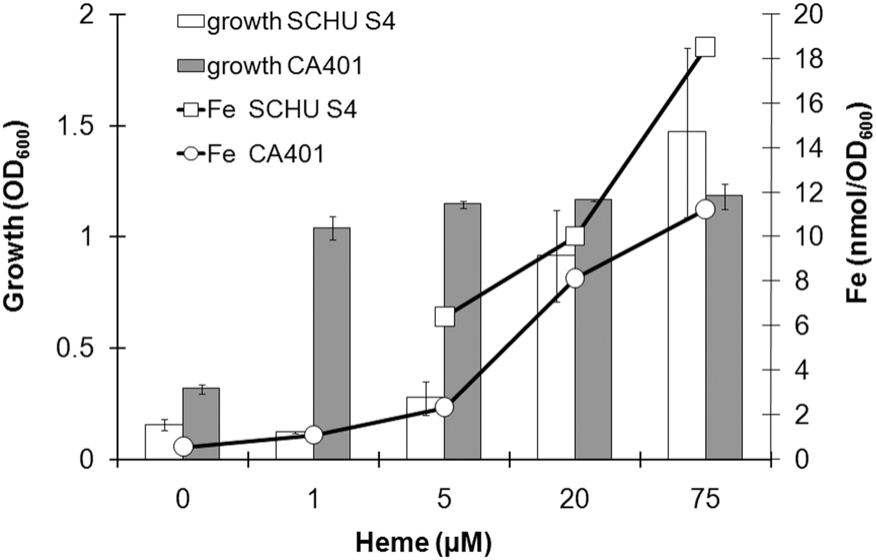
Growth response of SCHU S4 and CA401 in C-CDM to increasing heme concentrations and amounts of iron incorporated at the indicated heme concentration. Both strains were cultivated on DFO plates prior to the assays to reduce their internal storage of iron.

The capability of SCHU S4 to grow and to acquire iron from heme was compared to that of *V*. *cholerae* CA401, a strain with a well-characterized heme uptake apparatus [15,16]. The latter strain grew poorly in C-CDM without heme but already with the addition of 0.8 μM of heme, it reached an optical density at 600 nm (OD) of 1.0 within 5 h (Fig. 3). SCHU S4 did not grow in this concentration of heme and required 75 μM of heme and a 24 h incubation period to reach a similar density (Fig. 3). Despite the poor growth, SCHU S4 had acquired more iron than CA401 per OD after cultivation in 5, 20 or 75 μM of heme (*P* < 0.05) (Fig. 3). An OD of 1.0 corresponded to a similar density, 3 x 10^9^ CFU ml^-1^, for each strain.

Collectively, the data demonstrates that SCHU S4 rapidly binds heme and accumulates heme-bound iron at least as efficiently as CA401, which has a well-characterized heme-uptake system. However, equimolar concentrations of heme-derived iron did not support growth of SCHU S4 nearly as efficiently as did FeSO_4_ or as efficiently as it supported growth of CA401. This may indicate that heme-bound iron is not efficiently utilized by SCHU S4 and/or that the utilization of heme is highly energy-demanding.

### Screening of a Tn mutant library of SCHU S4 for utilization of heme

*F*. *tularensis* does not encode any homologues to components of the traditional heme-uptake system. Thus, to better understand the prerequisites for heme uptake of SCHU S4, a Tn mutant library of SCHU S4 was screened for growth on heme plates. To validate the assay for heme-utilization, the growth of SCHU S4 on various concentrations of heme was investigated. DFO was added to the plates since it chelates free iron and thereby minimizes residual free iron in the agar. The SCHU S4 strain did not grow in the absence of heme but reached the maximum density of 5 within 2 days with the addition of FeSO_4_. SCHU S4 required a minimum of 450 μM of heme to reach the maximum density within three days and this concentration was therefore used in the screen. Of 553 Tn mutants, 23 showed less efficient growth than SCHU S4 (Table 1 and S1 Table). In total, we identified Tn insertions in 19 open reading frames in the 23 mutants since four open reading frames each were targeted in two mutants. Mutants that grew poorly were further tested for growth in C-CDM with heme at a concentration of 300 μM and DFO at a concentration of 10 μg/ml. The majority of Tn mutants were to some degree growth inhibited in both assays although the absolute growth defect of some of the mutants varied between the two growth conditions. For example, mutants with insertions in *glpX*, *carB* or *glpE* showed no growth inhibition in C-CDM at either the 24 h or 48 h time point (Table 1). This discrepancy likely reflects that the role of a specific gene for heme utilization varies with the milieu that the bacteria reside in. Notably, all Tn mutants grew as well as SCHU S4 on agar and in C-CDM supplemented with 26 μM of FeSO_4_.

**Table 1:** Characterization of Tn mutant strains of SCHU S4.

Strain/Tn mutant	Agar + heme^a^		C-CDM + heme^b^		Heme sensitive
	3 days	6 days	24 h	48 h	
SCHU S4	+++++^c^	+++++	0.5 ± 0.06^d^	2.20 ± 0.07	no^e^
Tn-*FTT0134*	++	++++	0.14 ± 0.01	0.27 ± 0.05	No
Tn-*tet*	-	+	0.20 ± 0.02	1.7 ± 0.24	No
Tn-*FTT0454*	+	+++	0.15 ± 0.01	0.15 ± 0.01	Yes^f^
Tn-*FTT0609*	-	++	0.26 ± 0.01	0.88 ± 0.01	No
Tn-*elbB-15*	-	+	0.23 ± 0.01	0.45 ± 0.02	No
Tn-*elbB-10*	+	++++	0.20 ± 0.01	0.50 ± 0.07	No
Tn-*FTT0655*	-	-	0.26 ± 0.01	0.86 ± 0.05	NM^g^
Tn-*aroG*	+++	+++++	0.13 ± 0.01	0.10 ± 0.01	No
Tn- *pilA*	+	+++	0.26 ± 0.03	1.37 ± 0.09	no
Tn-*FTT1093c-17*	++	++++	0.36 ± 0.03	1.20 ± 0.20	no
Tn-*FTT1093c-18*	+	+++	0.38 ± 0.03	0.98 ± 0.09	no
Tn-*FTT1401–1*	++	++++	0.25 ± 0.04	0.94 ± 0.14	yes
Tn-*FTT1401–7*	++	++++	0.30 ± 0.01	1.43 ± 0.23	no
Tn-*wbtC-6*	-	+++	0.25 ± 0.05	2.12 ± 0.27	no
Tn-*wbtC-14*	-	+	0.22 ± 0.02	1.40 ± 0.25	no
Tn-*wbtE*	-	-	0.20 ± 0.03	1.25 ± 0.22	no
Tn-*wbtI*	-	+	0.28 ± 0.02	2.3 ± 0.06	no
Tn-*kdtA*	-	+	ND^h^	ND	NM
Tn-*rpmG*	+	+++++	0.32 ± 0.02	0.75 ± 0.02	no
Tn- *glpX*	-	+++++	0.5 ± 0.41	2.19 ± 0.25	no
Tn-*carB*	+++	++++	0.84 ± 0.28	2.34 ± 0.12	no
Tn-*tolC*	-	++	0.15 ± 0.05	1.20 ± 0.21	no
Tn-*glpE*	+	++++	0.41 ± 0.06	2.46 ± 0.10	no

^a^ A concentration of 450μM of heme was used.

^b^ A concentration of 300 μM of heme was used.

^c^ Growth was scored as—(no growth) or to a maximum of +++++

^d^ OD

^e^ >100% survival after exposure to 900 μM heme for 24 h at 37°C without rotation.

^f^ < 25% survival after exposure to 900 μM heme for 24 h at 37°C without rotation.

^g^ NM = not measurable, the strain did not survive in PBS at 37°C over night and therefore the survival could not be calculated.

^h^ ND = not determined.

Many studies have identified direct toxic effects of heme on various bacterial species [13,14]. Therefore, we investigated if this was the reason as to why the Tn mutants did not grow well. When exposed to a concentration of 900 μM of heme for 24 h in CDM, all of the SCHU S4 bacteria survived and so did the majority of mutants (Table 1), the exceptions were mutants with insertions in *FTT1401*, annotated to encode a prophage repressor protein, or *FTT0454*, encoding a protein belonging to the glycosyl transferase group 2 family. Thus, these genes appear not to be required for the utilization of heme, but rather to mitigate its potentially toxic effects.

Of the 16 open reading frames identified as required by *F*. *tularensis* to efficient utilize heme as an iron source, *tet* and *tolC* were the sole genes whose corresponding proteins are known to have a direct role in transport across the bacterial envelope. However, neither Tn-*tolC* nor Tn-*tet* acquired less iron than SCHU S4 when heme was the iron source as determined by the ferrozine assay (data not shown) and therefore it is unlikely that these proteins are part of a designated heme-uptake apparatus.

In summary, the screening of the Tn mutants on agar plates or in C-CDM with heme and the heme toxicity test identified 16 genes that were required by SCHU S4 to efficiently utilize heme and two of these genes were required to avoid heme toxicity. The screen did not identify any obvious components of a tentative heme-uptake apparatus and therefore we did not pursue any further studies of these genes.

### Requirements of heme-dependent growth of SCHU S4

**Fig 4:**
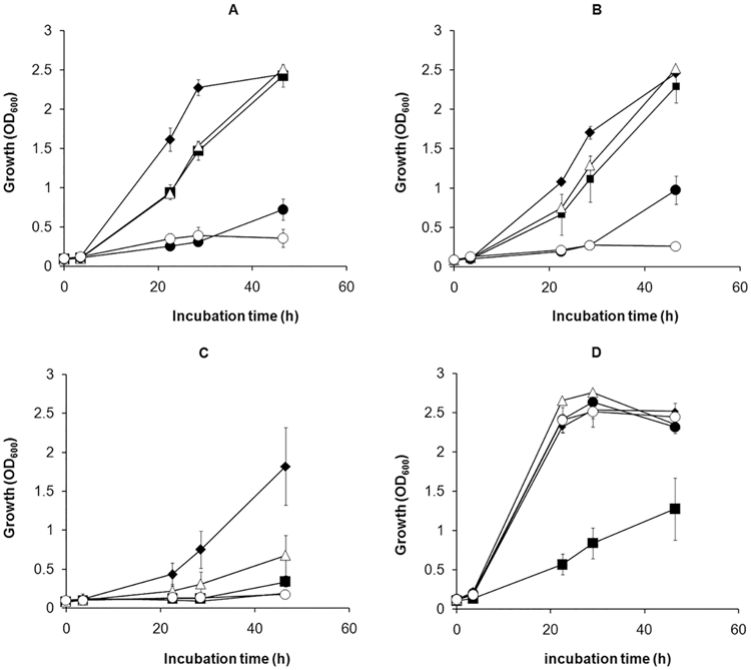
Growth of SCHU S4 (filled diamonds), Δ*feoB* (filled squares), Δ*fslA* (open triangles), ∆*fupA* (filled circles) and Δ*fslA*/∆*fupA* (open circles) in C-CDM supplemented with heme A) 150 μM B) 75 μM and C) 20 μM or FeSO_4_ D) 26 μM. All strains were iron depleted prior to the assay.

To better understand the prerequisites for heme uptake, we investigated the phenotypes of deletion mutants lacking expression of proteins previously shown to be directly or indirectly involved in iron uptake and utilization. To this end, we studied the phenotypes of deletion mutants lacking expression of FslA, necessary for the synthesis of the sole siderophore of *F*. *tularensis*, FeoB, which is necessary for efficient siderophore-independent iron uptake, and FupA, which has been shown to be necessary for effective siderophore-dependent and siderophore-independent iron utilization. In addition, we also included a double deletion mutant, Δ*fslA*/Δ*fupA*. None of the mutants grew as efficiently as did SCHU S4 in C-CDM when heme was the iron source (Fig. 4). The double mutant Δ*fslA*/Δ*fupA* was most compromised and exhibited minimal growth even with the highest concentration of heme (150 μM). Δ*fupA* grew slightly better than the double mutant but the growth rate and maximal density reached was much lower than for SCHU S4 at all concentrations tested. The growth rates of the Δ*fslA* and Δ*feoB* mutants were reduced at 75 and 150 μM of heme but these strains eventually reached the same maximal densities as SCHU S4. However, at the lowest concentration, 20 μM of heme, the maximal densities of these mutants were more than 1.0 OD_600_ lower than SCHU S4.

**Fig 5:**
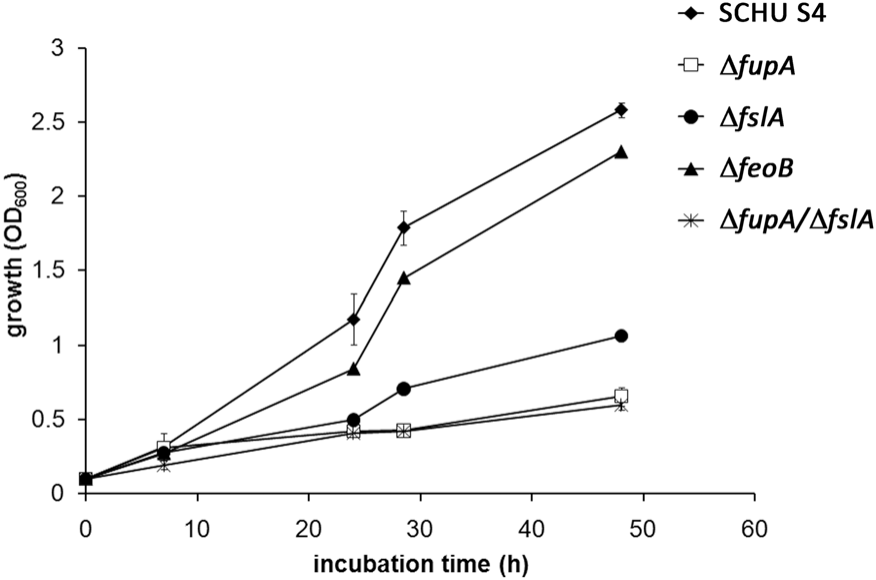
Growth of SCHU S4 (filled diamonds), Δ*feoB* (filled triangles), Δ*fslA* (closed circles), ∆*fupA* (closed circles) and Δ*fslA*/∆*fupA* (stars) in C-CDM supplemented with 300 μM heme and 10 μg/ml DFO. All strains were iron depleted prior to the assay.

Importantly, C-CDM did not support growth of any of the strains. Nevertheless, we wanted to investigate if residual iron in C-CDM affected the results and therefore we also studied growth of the strains in C-CDM supplemented with DFO to sequester any residual free iron in the medium. In C-CDM supplemented with 300 μM of heme and 10 μg/ml of DFO, the growth defect of the mutants was obvious, in particular marked deficiency was observed for Δ*fsl*A and Δ*fup*A and the double mutant Δ*fsl*A/Δ*fup*A (Fig. 5). However, all strains, including SCHU S4, showed reduced growth kinetics after the addition of DFO (Fig. 4 and 5). Thus, residual free iron, which *per se* is not sufficient to support growth of *F*. *tularensis*, is apparently capable to enhance the growth in the presence of heme (Fig. 4 and 5).

In summary, the results demonstrated that the *F*. *tularensis* siderophore system, FeoB, and FupA all contributed to the heme-dependent growth of SCHU S4 and there appeared to be a redundancy between the pathways, although FupA appeared to have the most crucial role. The heme-dependent growth pattern was distinct from the FeSO_4_-growth pattern, although, as expected, the growth of Δ*feoB* was compromised with the latter iron source as well.

### Evaluation of heme toxicity

Many studies have identified direct toxic effects of heme on various bacterial species [14]. Therefore, we investigated if this was the reason as to why Δ*fslA*, Δ*fupA*, Δ*fslA /*Δ*fupA* and Δ*feoB* showed impaired growth with heme as the iron source. All the mutants and SCHU S4 grew to a similar extent in C-CDM supplemented with 26 μM of FeSO_4_ or FeSO_4_ and 300 μM of heme (data not shown). These results show that heme *per se* does not impair the growth of any of the mutants.

### Iron incorporation during heme-dependent growth

To determine if the mutants were defective for iron uptake when heme was the iron source, samples were obtained from the cultures and analyzed by the ferrozine assay. After growth in the presence of 150 μM of heme for 24 h, Δ*feoB* contained 56 ± 2.4% of the amount of iron compared to SCHU S4 (*P* < 0.001), whereas Δ*fslA* contained similar amounts as SCHU S4 and Δ*fupA* and Δ*fslA*/Δ*fupA* contained 142 ± 9.3 and 142 ± 10%, respectively (*P* < 0.05). After growth in the presence of 20 μM heme, Δ*fslA* exhibited the most severe growth defect (Fig. 3C) and it had accumulated significantly less iron, 75 ± 3%, than SCHU S4 (*P* < 0.01).

**Fig 6:**
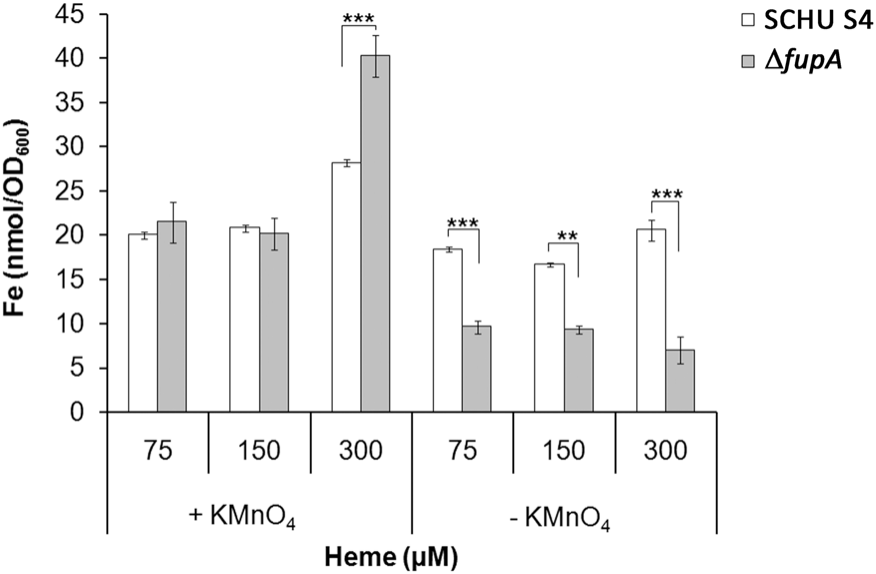
SCHU S4 (white bars), ∆*fupA* (grey bars) were cultivated in C-CDM supplemented with 75, 150 or, 300 μM of heme. When the cultures had reached an OD of at least 1.0, samples were withdrawn for analysis of incorporated iron by the ferrozine assay. One set of samples was treated with KMnO_4_, which mediates the release of low-molecular weight-bound iron (including heme-bound iron) and protein-bound iron. All strains were iron depleted prior to the assay.

In view of the impaired growth of ∆*fupA* and Δ*fslA*/Δ*fupA* with heme, it was unexpected that these mutants contained significantly more iron than did SCHU S4. The ferrozine assay involves a step where the bacterial lysate is treated with KMnO_4_ in order to release iron from macromolecules. In a parallel set of samples, this step was omitted in order to evaluate how much of the iron that constituted the free iron pool, *i*.*e*., the metabolically accessible proportion. Lower amounts of Fe were found in SCHU S4 without KMnO_4_ treatment vs. with treatment when cultivated in 150 or 300 μM of heme (*P* < 0.001 and *P* < 0.05, respectively), whereas the levels were similar at the two lowest concentrations of heme tested (Fig. 6). Regardless of heme concentration, the amount of iron detected in ∆*fupA* decreased markedly when the KMnO_4_ treatment was omitted and was lower than in samples of SCHU S4, (*P* < 0.001 for 150 and 300 μMl and *P* < 0.01 for 75 μM) (Fig. 6).

In summary, the growth defect of Δ*fslA* and Δ*feoB* with heme as iron source appeared to be due to impaired acquisition of iron, whereas ∆*fupA* incorporated at least as much iron as SCHU S4. The ferrozine assay omitting the KMnO_4_ step demonstrated; however, that a substantially larger portion of the iron in ∆*fupA* was sequestered to macromolecules, thereby most likely making it inaccessible for rapid utilization, which can explain the much impaired growth of the mutant.

### Expression of heme-utilization and *fsl* genes

By real-time PCR, it was analyzed how the genes identified in the Tn mutant screen, as well as *fupA*, *feoB*, and the siderophore operon genes, *fslA-F*, were regulated by SCHU S4 when cultivated on DFO agar (iron-depleted), or DFO agar supplemented with heme (designated heme plates). *fslA*, *B*, *C*, *E* and, *F*, which are induced in response to iron deficiency, showed significantly lower expression during growth on heme plates (Table 2). Expression of *feoB*, ∆*fupA* or Δ*fslD* was not affected by the culture conditions (Table 2) and none of the genes identified as related to heme utilization in the Tn-screen were found to be differently regulated by the culture conditions tested (data not shown).

**Table 2:** Transcriptional regulation of genes related to iron utilization in SCHU S4.

Gene number/name	Transcriptional regulation of genes^a^	
	DFO plate^b^	Heme plate
*FTT0029c*, *fslA*	220 ± 6.3	64 ± 8.3***
*FTT0028c*, *fslB*	76.4 ± 7.7	32.8 ± 5.9*
*FTT0027c*, *fslC*	43.0 ± 1.2	21.9 ± 4.4*
*FTT0026c*, *fslD*	6.0 ± 0.7	4.2 ± 0.9*
*FTT0025c*, *fslE*	4.0 ± 0.2	1.6 ± 0.2**
*FTT0024c*,*fslF*	4.2 ± 0.2	3.4 ± 0.1*
*feoB*	2.4 ± 0.51	2.6 ± 0.8
*fupA*	14.0 ± 1.79	12.1 ± 3.5

^a^ The expression of the genes was analyzed by quantitative real-time PCR. Results are expressed as RCN means ± SEM of triplicate samples from at least three independent samples.

^b^ The cDNA analyzed was synthesized from RNA prepared from SCHU S4 cultivated on the indicated plate overnight.

*P < 0.05 *vs*. gene expression on DFO plates.

**P < 0.01 *vs*. gene expression on DFO plates.

***P < 0.001 *vs*. gene expression on DFO plates.

## Discussion

Our study demonstrates that heme as the sole iron source supports rapid growth of *F*. *tularensis* at high concentrations. The prerequisites for effective heme utilization apparently are highly complex and the present data together with genomic information indicate that *F*. *tularensis* does not harbor any heme-acquisition system similar to those utilized by other Gram-negative bacteria. These heme-uptake systems described so far in other bacteria are similar and consist of a specific outer membrane receptor, a periplasmic heme transport protein, and an inner membrane ABC transport system [11]. Moreover, translocation of heme through the outer membrane normally requires energy generated by the TonB complex, but *F*. *tularensis* does not encode any homologues to components of the traditional heme-uptake systems or TonB. In the cytosol, heme is either directly incorporated into heme proteins or cleaved by heme oxygenase, which leads to the release of iron. Thus, again *F*. *tularensis* appears to be distinct since it relies on *FeoB*, which transports free ferrous iron into the cytoplasm to utilize heme as an iron source. The presence of a dechelatase in Gram-negative bacteria has been proposed as an alternative pathway for release of iron from heme, although the hypothesis has not generally been accepted [17]. However, there is no indication that *F*. *tularensis* possesses such dechelatase activity. It should be noted that the transposon library screened did not nearly represent the whole *F*. *tularensis* genome and therefore it is still possible that it may contain genes of direct relevance for heme uptake.

Compared to *V*. *cholerae*, SCHU S4 required high concentrations of heme to grow rapidly. This finding may be a reflection of a lack of a traditional heme-uptake system in *F*. *tularensis* and is in agreement with our findings of the screening of the transposon mutant library that failed to identify any genes encoding obvious heme-uptake proteins. However, the inefficient growth using heme was not related to inefficient iron uptake since intracellular iron was higher in *F*. *tularensis* at comparable heme concentrations. In addition, the majority of the genes of the *fsl*-operon including *fslE*, encoding the receptor for ferric siderophores was down-regulated in SCHU S4 grown on heme plates, indicating iron homeostasis. Previously, it has been found that *fslE* is tightly regulated by iron availability and is not induced under conditions of modest iron deprivation [3,18]. Thus, the low expression level of *fslE* in response to heme indicated that SCHU S4 was capable to acquire sufficient amounts of iron. Nevertheless, the requirement of high concentrations of heme for rapid growth may reflect that heme-derived iron is not available intracellularly to support effective growth or that the in *vitro* system does not effectively trigger the signal for optimal gene expression with regard to heme utilization.

*F*. *tularensis* has been shown to be able to utilize ferric and ferrous iron, transferrin and lactoferrin as iron sources [10,19]. This study adds heme to the repertoire of SCHU S4. Since heme is the most abundant source of iron in the human body and it has been shown to be essential for virulence of many pathogenic bacteria, our observation that *F*. *tularensis* can utilize heme for efficient growth is of general relevance. Often, heme is acquired via specific hemolysis, but although *F*. *tularensis* does not produce a hemolysin, it still encounters heme or hemoglobin at both intracellular and extracellular sites of infection, *e*.*g*., release of free heme may result from degradation of cells at sites of infection. Also, heme may become accessible through degradation in liver or spleen of hemoglobin-haptoglobin complexes scavenged from the circulation [12].

We found that the systems utilized by *F*. *tularensis* for acquisition of other forms of iron, siderophores, FeoB and FupA, all contributed to the heme utilization. FupA, which is an outer membrane protein, facilitates the uptake of ferrous iron and also of the siderophore-bound iron [9,10]. We here observed that ∆*fupA* contained higher amounts of intracellular iron than did SCHU S4, demonstrating that insufficient iron uptake was not the reason for the poor growth of the mutant on heme-containing media. The modified ferrozine assay demonstrated; however, that the free iron pool was markedly diminished. This observation is in accordance with our previously reported finding that ∆*fupA* is highly resistant to streptonigrin [9], which is considered to be a measure of a small free intracellular iron pool [20,21]. Based on this previous and the present observations, it appears that the poor growth of ∆*fupA* probably is not related to heme utilization *per se* but rather to the role of FupA to maintain an iron pool that is easily accessible to effectively support growth.

A recent study demonstrated that the siderophore system and the Feo system are the sole iron uptake mechanisms in LVS [5]. In line with this, the results of the present study show that the utilisation of heme by SCHU S4 also relies on these two systems. In *vivo*, the iron source(s) may vary depending on the niche where the bacterium resides. Our finding of an enhanced growth response of *F*. *tularensis* to heme in the presence of residual free iron, which *per se* was not sufficient to support growth is likely highly relevant to the *in vivo* situation. As aforementioned, *F*. *tularensis* likely encounters heme at several different niches and, in addition, simultaneously also other iron source such as ferric and ferrous iron, transferrin and lactoferrin. Thus, *in vivo F*. *tularensis* may utilize several different iron sources simultaneously to grow optimally.

The growth of SCHU S4 even at high concentrations of heme demonstrates that it harbors effective mechanisms to resist the toxicity. This is likely an important virulence trait since bacteria encounter heme and heme-containing proteins at high concentrations in *vivo*. The resistance was found to be dependent on YfhD and FTT1401. The former is a group 2 glycosyl transferase, which contributes to the virulence of numerous pathogens either by contributing to the synthesis of a polysaccharide capsule or the LPS [22]. Previous studies have demonstrated that its adds galactosamine-1-phosphate to the lipid A of *F*. *tularensis* [23,24]. This type of modification is likely to alter the negative charge of the lipid A [24] and such an alteration in other Gram-negative bacteria enhances the resistance to polymyxin and cationic antimicrobial peptides [25]. Based on this knowledge, we hypothesize that the heme-sensitive phenotype of Tn-*yfhD* may be due to the absence of the galactosamine-1-phosphate modification of lipid A. FTT1401 is a homologue of LexA (locus for X-ray sensitivity A), which, together with RecA (recombinase A), are key regulators of the SOS system, which is essential for DNA repair [26]. There is only limited information available regarding the *F*. *tularensis* SOS response, but RecA from *F*. *novicida* was found to perform DNA repair in *E*. *coli* [27]. In view of the highly conserved nature of the SOS system, it is likely that the heme-sensitive phenotype of the *FTT1401* mutant is indeed related to DNA damage induced by exposure to heme.

In summary, the present study demonstrates that *F*. *tularensis* efficiently tolerates the toxicity of heme and that it can utilize heme for rapid replication. The majority of genes identified as important for these traits has previous been reported to be virulence factors and this emphasizes the importance of heme utilization/tolerance to the virulence of *F*. *tularensis*.

## Materials and Methods

### Bacterial strains

The *F*. *tularensis* subspecies *tularensis* strain SCHU S4 was obtained from the *Francisella* Strain Collection (FSC), Swedish Defence Research Agency, Umeå. The construction of the transposon (Tn) library used has been published elsewhere [28]. The construction of the deletion mutants Δ*fslA* and ∆*fupA* as well as ∆*fupA* complemented in *trans* has been described elsewhere [9]. The *V*. *cholerae* CA401 strain was a kind gift from Dr Shelley Payne, University of Texas at Austin.

The Δ*feoB* mutant was generated by allelic replacement essentially as described [28]. Briefly, the fragments located upstream or downstream of the gene were amplified by PCR and a second overlapping PCR using purified fragments 1 and 2 as templates was performed. After restriction enzyme digestion and purification, PCR fragments were cloned to the pDMK2 vector. The resulting plasmids were first introduced to *E*. *coli* S17–1 and then transferred to SCHU S4 by conjugation. Clones with plasmids integrated into the chromosome by a single recombination event were selected on plates containing kanamycin and polymyxin B. Integration was verified by PCR. Clones with integrations were then subjected to sucrose selection. This procedure selected for a second cross-over event in which the integrated plasmid, encoding *sacB*, was excised from the chromosome. Kanamycin-sensitive, sucrose-resistant clones were examined by PCR confirming the deletion of the genes. All primers sequences and detailed descriptions of the construction of the plasmids used to generate mutants are available upon request All bacteriological work was carried out in a biosafety level 3 facility certified by the Swedish Work Environment Authority.

### Preparation of growth media

Chamberlain’s defined medium (CDM) without FeSO_4_ [29] was iron-depleted by chelation (C-CDM) as described previously [9]. Briefly, 1% (w/v) of Chelex-100 (Bio-Rad, Hercules, CA, USA) was added and the mixture was rotated for 24 h at 4°C. The Chelex-100 was removed by filtering the medium through a 0.45 μm Millipore filter and the chelating step was repeated once (Biosciences, Stockholm, Sweden). The medium was thereafter supplemented with essential cations (MgSO_4_ 0.55mM, ZnCl_2_ 1.4 μM, CuSO_4_ 0.2 μM, MnCl_2_ 1.0 μM, and CaCl_2_ 5 μM) and sterilized by filtration through a 0.2-μm-pore-size Millipore filter.

Deferoxamine (DFO) (Sigma-Aldrich, St. Louis, USA) was added to prepare agar plates devoid of free iron in the medium (DFO plates). These plates were composed of 1 part of 4% GC II agar base (BD Diagnostic Systems, Sparks, MD), 1 part of CDM without FeSO_4_, and 25 μg/ml DFO. The designation heme plates was used for the DFO plates supplemented with various concentrations of Porcine heme (Sigma-Aldrich).

### Screening of a SCHU S4 Tn library for growth with heme as the sole iron source

In total, 553 Tn mutants in SCHU S4 were screened for growth on agar plates with heme. It is estimated that these clones represent a coverage of 25.4% of the whole genome. Bacteria cultivated overnight on MC plates were re-cultivated overnight on DFO plates. Bacteria cultivated by this procedure were thereby effectively depleted of their internal iron pool and will henceforth be designated iron-depleted bacteria. The iron-depleted bacteria were resuspended to an optical density at 600 nm (OD) of 1.0 in PBS. Bacteria from 1 ml were collected by centrifugation at 13,000 rpm for 5 min. The bacterial pellet was resuspended in 1 ml of PBS. 2.5 μl of the bacterial suspension was dropped onto DFO plates with 450 or 900 μM of heme (Sigma-Aldrich) (four replicates of each strain). Each strain was also tested for growth on a DFO plate (negative control) and a plate with 26 μM FeSO_4_ without DFO (positive control). The plates were incubated at 37°C in 5% CO_2_ and monitored for growth after 3 and 6 days. The growth zones were scored from 0 to 5 with the following criteria: 0; no growth, 1; thin growth at the edge of the bacterial droplet, 2; thin growth at the edge and the middle of the bacterial droplet, 3; moderate growth at the edge of the droplet and thin growth in the middle of the droplet, 4; moderate growth at the edge and the middle of the bacterial droplet, 5; thick growth at the edge and the middle of the bacterial droplet.

### Growth in C-CDM with FeSO_4_ or heme

Iron-depleted bacteria were resuspended to an OD of 1.0 in C-CDM. The bacterial suspension was washed once by collecting the bacteria by centrifugation at 13,000 rpm for 5 min and the resulting bacterial pellet was resuspended in C-CDM to an OD of 1.0. The bacterial suspension was 10-fold diluted into C-CDM supplemented with various concentrations of heme, or 26 μM of FeSO_4_. CDM without any supplements were used as a negative growth control. When indicated the cultures were supplemented with 10 μg/ml DFO (Sigma). The tubes were incubated at 37°C with 200 rpm agitation and the OD was measured at indicated time points.

### Heme toxicity test

The potential toxicity of heme was assessed by use of two assays. In the first assay, iron-depleted bacteria were resuspended to an OD of 1.0 in C-CDM, diluted 1,000-fold in C-CDM or in C-CDM with 900 μM of heme (triplicates of each strain and condition). The tubes were incubated for 4 or 20 h at 37°C without rotation. At the end of the incubation period, the samples were serially diluted in PBS and spread on MC plates that were incubated at 37°C in 5% CO_2_ for 3 days before enumeration of the CFU. In the second assay, iron-depleted bacteria where resuspended to an OD of 1.0 in C-CDM and diluted 10-fold with C-CDM supplemented with 300 μM of heme, 26 μM of FeSO_4_, or both iron sources. The tubes were incubated at 37°C with 200 rpm agitation and the OD was measured at 6, 24 and 48 h.

### Real-time PCR analysis of gene expression

SCHU S4 was transferred from MC plates and cultivated overnight on DFO plates or heme plates (450 μM of heme) in 37°C, 5% CO_2_. DFO plates are devoid of free iron and heme is the sole iron source in heme plates. The bacteria from each plate were thereafter resuspended to an OD of 1.0 in PBS and 1 ml of the bacterial suspension was centrifuged for 5 min at 13,000 rpm. RNA was extracted from the bacterial pellet using Trizol according to the manufacturer’s protocol (Invitrogen). cDNA was synthesized from this RNA and quantitative real-time PCR (RT-PCR) was used to analyze the cDNA samples. In order to remove contaminating DNA, the RNA samples were DNase-treated (DNA-*free* kit, Ambion, Inc, Austin, TX, USA) in accordance with the protocol supplied by the manufacturer. The RNA was quantified by Nanodrop (Thermo Fisher Scientific, Wilmington, DE, USA). cDNA was synthesized from 1 μg of the extracted RNA using RNIscript (BioRad) according to the protocol provided by the manufacturer. A negative control was prepared by excluding the enzyme from the cDNA synthesis (control 1). The cDNA samples were thereafter diluted and stored at -20°C.

RT-PCR was performed in the ABI Prism 7900HT Sequence Detection System (Applied Biosystems, Foster City, CA, USA) using the SYBR green I PCR kit (Applied Biosystems) as recommended by the manufacturer. Each reaction contained 12.5 μl of the SYBR green mix, 250 nM of forward and reverse primers, 5 μl of a cDNA and the total volume was adjusted with water to 25 μl. Forward and reverse primers were obtained from Invitrogen and their sequences can be obtained upon request. The reactions were performed in MicroAmp 96-well plate (Applied Biosystems) capped with MicroAmp optical adhesive seal. The reactions were incubated at 50°C for 2 min, 10 min at 95°C followed by 45 cycles of 15 s at 95°C and 1 min at 60°C. The final cycle consisted of incubation at 95°C for 15 s, 60°C for 15 s, and at 95°C for 15 s. To control for contaminating DNA in the reaction, tubes with template from control 1 (see above) and tubes with water instead of template were included in the analysis. The controls gave Ct values (Ct is the threshold cycle) below detection level or at least 8 cycles later than the corresponding cDNA. Relative copy numbers (RCN) of the mRNA of selected genes were expressed in relation to the expression of the housekeeping gene *tul4* [30] and calculated according to the following equation: RCN = 2^-ΔCt^ × 100 where ΔCt is Ct_(target)_-Ct_(*tul4*)_ [31]. Thus, the copy number of a given gene is related to the copy number of *tul4*. Normalized Ct-values were used for statistical evaluation of the data.

### Liquid heme-binding assay

The liquid heme-binding assay measures the ability of whole bacteria to bind heme and was performed essentially as previously described [32]. Briefly, iron-depleted SCHU S4 was suspended in PBS to an OD of 1.0 and collected by centrifugation at 10, 000 × g for 5 min. The resulting pellet was resuspended in 1 ml 0.1 M TRIS, pH 8.0 and this washing step was repeated once before the bacteria were finally resuspended in 800 μl of C-CDM. 200 μl C-CDM supplemented with heme to give a final concentration of heme of 300, 150 or 75 μM was added to the bacterial suspension. Control tubes containing C-CDM with corresponding concentrations of heme but without bacteria was prepared. After 1 h incubation at 37°C and 200 rpm, the bacteria were pelleted by centrifugation at 10,000 × g for 5 min and the resulting supernatant was collected to determine the absorbance at 400 nm (A_400_). The extent of the hemin binding is a reflection of the reduction in the A_400_ of the test tube relative to the corresponding control. The concentration of heme bound by the bacteria was calculated from a standard curve generated by serial dilution of heme in C-CDM.

### Ferrozine assay

A ferrozine-based method was used to measure the total amount of iron in the bacterial samples [33]. Ferrozine forms a complex with Fe^2+^ that absorbs strongly at 562 nm. Iron-depleted bacteria were resuspended to an OD of 1.0 in C-CDM and diluted 10-fold into C-CDM supplemented with indicated concentrations of heme or FeSO_4_. The tubes were incubated at 37°C with 200 rpm agitation and samples corresponding to 3 × 10^9^ CFU were withdrawn from the cultures at indicated time points and collected by centrifugation at 13,000 rpm for 5 min. The resulting bacterial pellet was washed one time in 1 ml 10 mM Tris pH 8.0. Pelleted bacteria were lysed with 100 μl of 50 mM NaOH. The solution was mixed thoroughly and incubated in room temperature for at least 0.5 h to ensure complete lysis of the bacteria. One hundred μl of 10 mM HCl was added to the lysate. To release low-molecular weight-bound (including heme-bound iron) and protein-bound iron, the samples were treated with 100 μl of a freshly prepared solution of 0.7 M HCl and 2.25% (w/v) KMnO_4_ in H_2_O and incubated for 2 h at 60°C. Importantly, heme is not detected by the assay. When indicated, the KMnO_4_ treatment was omitted. All chemicals used were from Sigma-Aldrich. Thereafter, the samples were mixed with 100 μl of the iron detection reagent composed of 6.5 mM ferrozine, 6.5 mM neocuproine, 2.5 M ammonium acetate, and 1.0 M ascorbic acid dissolved in water. The samples were incubated for 10 min and insoluble particles were removed by centrifugation. Two hundred μl of the supernatant was transferred to a 96-well plate and the A_562_ nm determined in a microplate reader (Paradigm, Beckman Coulter, Bromma, Sweden). The iron content of the sample was calculated by comparing its absorbance to that of a range of samples with FeCl_3_ corresponding to Fe concentrations in the range of 0–180 μM of Fe that had been prepared identically to the test samples. The detection limit of the assay was 2.5 μM of Fe.

### Statistical evaluation

Welch’s *t* test was used to compare means between groups and Spearman’s rank correlation was used to study dependences between variables. All statistical analyzes were conducted using SPSS, version 20.

## Supporting Information

S1 Table Tn mutant strains identified as defective for growth on agar with heme. (DOCX)
