# Supplementary material for: Mechanisms of Heme Utilization by Francisella tularensis
Source: PLoS One. 2015 Mar 10;10(3):e0119143. doi: 10.1371/journal.pone.0119143 (PMC4355490; doi:10.1371/journal.pone.0119143)
Supplement: S1 Table — (DOCX) [file pone.0119143.s001.docx]

Table S1. Tn mutant strains identified as defective for growth on agar with heme

| Designation | Gene target | Insertion-location^a^ | Gene product | Virulence^b^ |
| --- | --- | --- | --- | --- |
| Tn-*FTT0134* | *FTT0134* | 91 | hypothetical membrane protein | [[34](#_ENREF_34)] |
| Tn-*tet* | *FTT0444* | 73 | multidrug transporter | [[35](#_ENREF_35)] |
| Tn-*yfdH* | *FTT0454* | 78 | glycosyl transferase, group 2 family protein | [[34](#_ENREF_34)] |
| Tn-*FTT0609* | *FTT0609* | 69 | Peptidase, M24 family protein | [[28](#_ENREF_28)] |
| Tn-*elbB-10* | *FTT0654* | 31 | DJ-1/PfpI family protein | [[35](#_ENREF_35),[36](#_ENREF_36)] |
| Tn-*elbB-15* | *FTT0654* | 67 | DJ-1/PfpI family protein |  |
| Tn-*FTT0655* | *FTT0655* | 34 | conserved hypothetical protein |  |
| Tn-*pilA* | *FTT0890c* | 27 | Type IV pilus fiber building block protein | [[37](#_ENREF_37),[38](#_ENREF_38),[39](#_ENREF_39)] |
| Tn-*aroG* | *FTT0963c* | 76 | Phospho-2-dehydro-3-deoxyheptonate aldolase | [[35](#_ENREF_35)] |
| Tn-*talA-17* | *FTT1093c* | 31 | Transaldolase |  |
| Tn-*talA-18* | *FTT1093c* | 30 | Transaldolase |  |
| Tn-*FTT1401-1* | *FTT1401* | 18 | prophage repressor protein |  |
| Tn-*FTT1401-7* | *FTT1401* | 37 | prophage repressor protein |  |
| Tn-*wbtI* | *FTT1455c* | 45 | sugar transamine/perosamine synthetase | [[28](#_ENREF_28),[40](#_ENREF_40)] |
| Tn-*wbtE* | *FTT1460c* | 74 | UDP-glucose/GDP-mannose dehydrogenase | [[34](#_ENREF_34)] |
| Tn-*wbtC-6* | *FTT1462c* | 31 | UDP-glucose 4-epimerase | [[41](#_ENREF_41)] |
| Tn-*wbtC-14* | *FTT1462c* | 7 | UDP-glucose 4-epimerase |  |
| Tn-*kdtA* | *FTT1561* | 1 | 3-Deoxy-D-manno-octulosonic-acid transferase | [[28](#_ENREF_28)] |
| Tn-*rpmG* | *FTT1604* | 33 | 50S ribosomal protein L33 |  |
| Tn-*glpX* | *FTT1631c* | 5 | GlpX protein | [[28](#_ENREF_28),[34](#_ENREF_34),[35](#_ENREF_35),[36](#_ENREF_36)] |
| Tn-*carB* | *FTT1664* | 55 | Carbamoyl-phosphate synthase large chain | [[34](#_ENREF_34)] |
| Tn-*tolC* | *FTT1724c* | 21 | outer membrane protein tolC precursor | [[28](#_ENREF_28)] |
| Tn-*glpE* | *FTT1748* | 62 | thiosulfate sulfurtransferase | [[35](#_ENREF_35)] |

^a^ Expressed as % of the total gene length calculated from the 5´ end.

^b^ Publications demonstrating a role of the target gene for the virulence of *F. tularensis* in mice.
